# Gastric Schwannoma: A Case Report and Literature Review

**DOI:** 10.7759/cureus.24785

**Published:** 2022-05-06

**Authors:** Sohaib Khan, Nagaraj Sanchitha Honganur, Sunil Kumar, Stephanie Lucas, Paula Dionisio

**Affiliations:** 1 Internal Medicine, Parkview Medical Center, Pueblo, USA; 2 Gastroenterology, Parkview Medical Center, Pueblo, USA

**Keywords:** spindle cell mesenchymal tumor, gastrointestinal stromal tumors, immunochemistry, fine-needle aspiration, muscularis propria, esophagogastroduodenoscopy, submucosal tumor, schwann cell, auerbach's plexus, gastric tumor

## Abstract

Gastric schwannomas (GS) are very rare spindle cell, submucosal mesenchymal tumors that arise from Schwann cells of nerve plexuses in the stomach wall. They are usually benign but can become malignant and metastasize to other organs. Surgical resection with biopsy is the gold standard for diagnosis and management of GS. In this article, we present a 68-year-old female patient who presented with abdominal pain, nausea, vomiting, and belching for a couple of months. Upon further evaluation, she was found to have a 4.2 cm gastric mass, which was consistent with gastric schwannoma through biopsy and immunohistochemistry. The patient underwent complete surgical resection of the tumor without any complications. In this article, we will discuss the literature about GS including its clinical presentation, diagnosis, and management options.

## Introduction

There are several spindle cell mesenchymal tumors that arise from the gastrointestinal (GI) tract and include leiomyoma, leiomyosarcoma, GI stromal tumors (GIST), and schwannoma. GIST is the most common mesenchymal tumor of the GI tract whereas schwannomas are rare and represent 2-6% of all mesenchymal tumors [1]. First explained by Verocay in 1910, schwannomas are benign, slow-growing tumors that can arise from nerves having Schwann cell sheath [2,3,4]. They arise commonly from the extremities, head, and neck but are very rare in the GI tract. The stomach is the most common site of involvement, constituting 60-70% of all cases involving the GI tract while the colon and rectum represent only 3% of the cases [1,3,4]. Constituting only 4% of all benign and 0.2% of all gastric tumors, gastric schwannoma (GS) is a rare submucosal tumor that develops from Schwann cells of the Auerbach's plexus of the stomach and was first mentioned by Daimaru et al. in 1988 [1,2,4,5]. If symptomatic, patients can experience abdominal pain, change in bowel habits, nausea, and vomiting [1,3,4]. Although imaging and upper endoscopy are suggested, accurate diagnosis and management are usually done with the help of surgical excision and biopsy along with immunohistochemistry, which is positive for S-100 but negative for c-Kit, smooth muscle actin, CD117, and CD34 [2,3]. Here we present the case of a 68-year-old female who was diagnosed with GS.

## Case presentation

**Figure 1:**
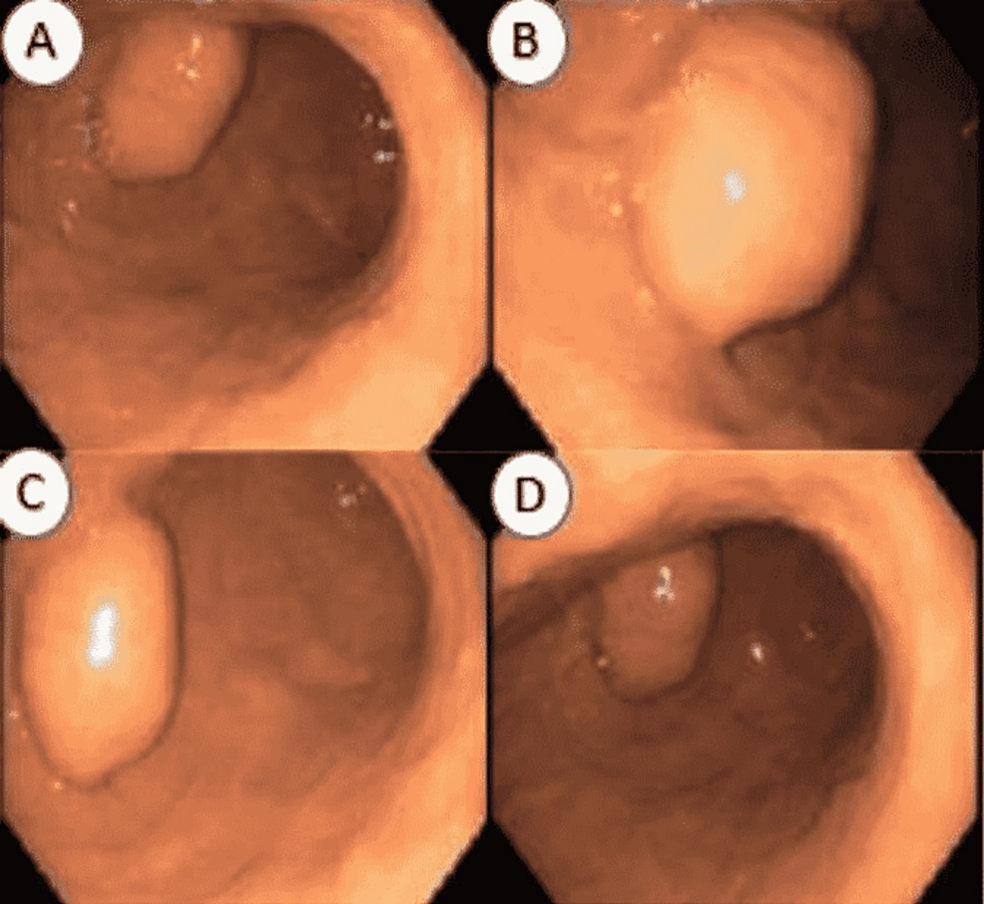
Esophagogastroduodenoscopy showing a submucosal mass in the gastric antrum (A-D).

A 68-year-old female, with an extensive history of multiple abdominal surgeries, visited her gastroenterologist because of nonspecific symptoms including abdominal pain, belching, abdominal distension, nausea, and occasional vomiting for the past couple of months. A recent computed tomography (CT) scan that was ordered for concerns of nephrolithiasis showed a 4.2 cm enhancing mass within the stomach along with prominent vascularity. The patient subsequently underwent an esophagogastroduodenoscopy (EGD) (Figure 1) and endoscopic ultrasound (EUS) that showed an intramural (subepithelial) and submucosal mass in the body of the stomach, which appeared to originate from within the muscularis propria.

**Figure 2:**
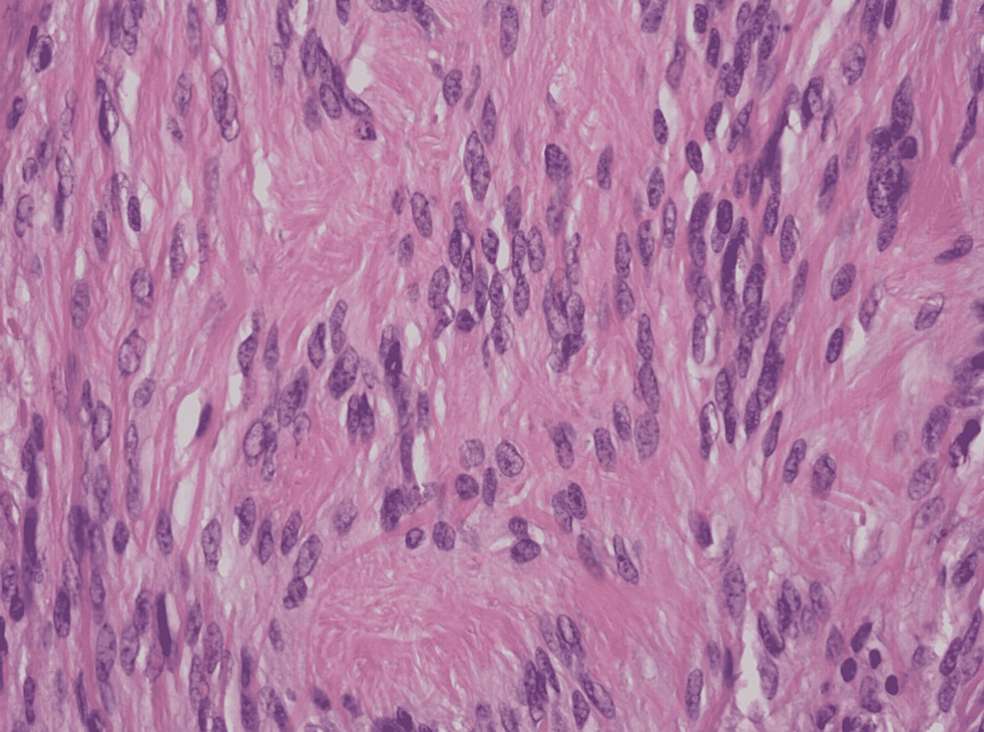
H&E stain at 400x showing neoplastic spindle cells exhibiting nuclear palisading and Verocay body formation. H&E: hematoxylin and eosin

**Figure 3:**
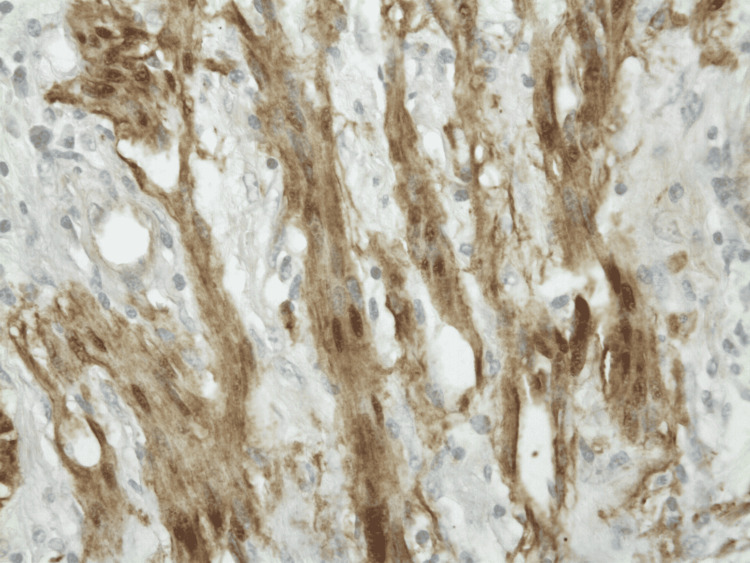
S-100 protein immunohistochemical stain at 400x showing cytoplasmic and nuclear staining of tumor cells.

**Figure 4:**
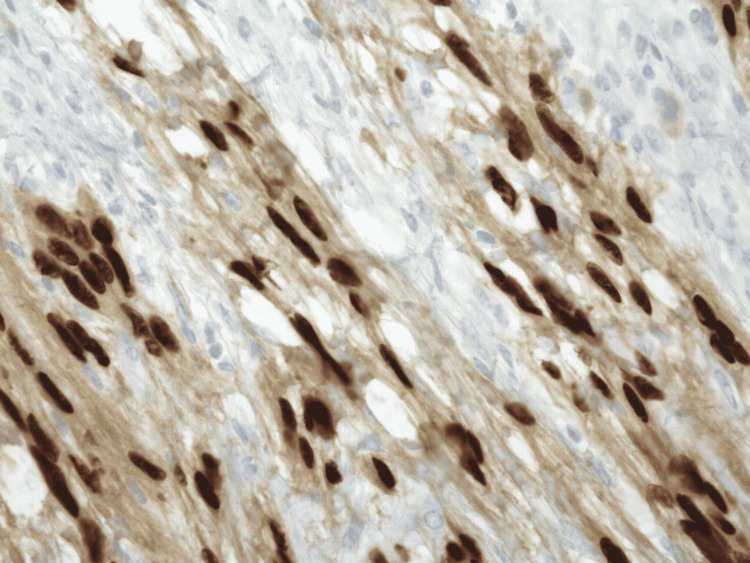
SOX10 immunohistochemical stain at 400x showing strong nuclear staining of tumor cells.

**Figure 5:**
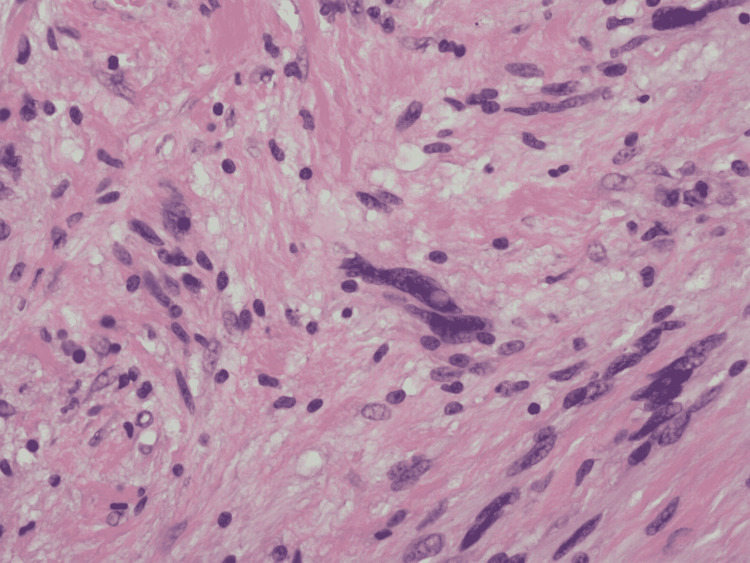
H&E stain at 400x showing focally marked nuclear pleomorphism/atypia, which may be observed in these tumors. Mitotic figures and necrosis are absent. H&E: hematoxylin and eosin

Biopsy of the mass showed a neural sheath tumor consisting of spindle cells with mild atypia, negative for CD117, smooth muscle actin, CD34, and positive for S100 (Figures 2, 3, 4, 5). This was consistent with gastric schwannoma (GS). The patient was referred to general surgery for further evaluation. While planning for surgical management, the patient underwent a CT scan of the chest and abdomen for metastatic workup. Chest CT did not show any evidence of metastatic disease but CT abdomen and pelvis showed gastro-hepatic ligament lymphadenopathy. The patient underwent a gastrectomy with Roux-en-Y reconstruction along with extensive lysis of adhesions. During the surgery, a large gastric mass was seen near the greater curvature of the stomach in the anterior aspect. The mass was sent for biopsy along with adjacent lymph nodes and the results were again consistent with GS and benign lymph node hyperplasia. There were no major intraoperative or postoperative complications and the patient had a very good recovery. She was eventually discharged on postoperative day (POD) nine with the advice of follow-up in the outpatient clinic for further evaluation.

## Discussion

First explained by Daimaru et al. in 1988, GS are rare, slow-growing, submucosal tumors that originate from Schwann cells of Meissner's or Auerbach's plexus of the stomach wall [1,2,3,4,6,7]. They constitute only 0.2% of all gastric tumors and are often confused with GISTs due to their similar presentation [3,6,7]. They are located in the submucosa or muscularis as solitary lesions and arise mostly from the stomach body (50%) followed by antrum (32%) and fundus (18%). Their size can vary from 0.5 cm to 15.5 cm in diameter. Upon dissection, they present as a yellow, gray, pearly white, and sometimes variegated mass in case of necrosis, hemorrhage or fatty infiltration [5,8]. In our patient, GS was found to originate from the muscularis propria in the body of the stomach.

Although GS are mostly benign, malignant transformation or primary malignant schwannoma can be seen in rare cases [3,6]. If the tumor is malignant, it does show a high Ki-67 proliferative index. Other features that indicate malignancy include size of the tumor, definite integrity, appearance of surrounding organs, hemorrhagic changes, and metastatic disease [1,6,8].

Clinically, GS are mostly asymptomatic and are usually found incidentally. As in our patient, when symptomatic, they can present with abdominal pain, abdominal fullness, change in bowel habits, nausea, vomiting, hematemesis, melena and even with intussusception. They can also compress surrounding structures leading to ulceration and bleeding [1,5,6].

Various modalities are available for the diagnosis of GS. These include magnetic resonance imaging (MRI), CT scan, EGD, and EUS with biopsy, each having its own diagnostic features. MRI can help to assess the tumor architecture, identify its exact location, and comment on its surroundings. CT scan can identify GS as an exophytic homogenous mass originating from the gastric wall. Endoscopically, GSs appear as a protruding submucosal mass with or without overlying ulceration. However, due to its submucosal location and minimal mucosal abnormality, endoscopic biopsies are mostly inconclusive as compared to EUS with fine needle aspiration (FNA) biopsy which can assist in preoperative evaluation of the tumor. Despite availability of all these modalities, the definite diagnosis of GS is made with biopsy after surgical excision of the tumor that displays an exophytic, demarcated lesion with a rubbery texture and without ulceration or calcification [1, 4, 5, 6].

Under the microscope, GS appear as firm, well-circumscribed, encapsulated tumors with a spherical, ovoid or multilobed configuration, developing near the nerves without their invasion [5,8]. On histologic examination, GS shows a fascicular arrangement with spindle-shaped nuclei. No necrosis, nuclear polymorphism, or mitosis is seen. As they are easily confused with GIST due to similar histologic presentation, immunochemistry staining is used to differentiate between the two tumors. GS shows positive staining for S100, vimentin, Leu-7, glial fibrillary acidic protein (GFAP), myelin-associated glycoprotein, and nonspecific enolase while desmin, CD117, striated muscle actin, smooth muscle actin, and myosin stain negative on immunohistochemistry [1,6,8]. Peritumoral lymphoid cutoff is another feature that helps to differentiate GS from GIST [9].

Various surgical options are available for the management of GS and can be approached via laparoscopy or laparotomy. Depending on the location, extent, and size of the tumor, it can be managed via en bloc resection, wedge resection, subtotal, near-total, or total gastrectomy. Despite these options, complete surgical excision of the tumor is considered as the gold standard for the management of GS. Endoscopic resection is another treatment option that have been described recently but is limited by the tumors > 3 cm and location within the muscularis propria as it usually requires surgical excision because of increased risk of perforation [6,8,9,10]. This was the case in our patient who underwent complete surgical resection due to tumor size of 4.2 cm and location in the muscularis propria. Chemotherapy and radiation can be used for malignant tumors after surgical excision [3,4,5]. With complete surgical excision, GS have a very good prognosis with a low rate of recurrence and, therefore, follow-up imaging studies are not usually recommended [11]. 

## Conclusions

GS are rare spindle-shaped mesenchymal tumors that originate from the Schwann cells of Meissner’s or Auerbach’s plexus present in the stomach wall. They are mostly benign and asymptomatic although sometimes patients can present with symptoms including abdominal pain, fullness, nausea, vomiting, change in bowel habits as seen in our patient. Diagnosis is usually made with biopsy and immunohistochemical staining after surgical resection of the tumor along with CT, MRI, and endoscopic biopsy. Treatment is mainly surgical resection along with chemotherapy and radiotherapy for malignant and metastatic disease. Follow-up is usually not required due to the low recurrence of the tumor. 
